# Strain and ligand effects in Pt-Ni alloys studied by valence-to-core X-ray emission spectroscopy

**DOI:** 10.1038/s41598-021-93068-0

**Published:** 2021-07-01

**Authors:** Jiatang Chen, Y. Zou Finfrock, Zhiqiang Wang, Tsun-Kong Sham

**Affiliations:** 1 Department of Chemistry, University of Western Ontario, London, ON N6A 5B7 Canada; 2 CLS@APS Sector 20, Advanced Photon Source, Argonne National Laboratory, Lemont, IL 60439 USA; 3 Science Division, Canadian Light Source Inc, Saskatoon, SK S7N 2V3 Canada

**Keywords:** Energy, Materials chemistry, Physical chemistry, Condensed-matter physics, Materials for energy and catalysis

## Abstract

Experimental detection of the Pt 5d densities of states in the valence band is conducted on a series of Pt-Ni alloys by high energy resolution valence-to-core X-ray emission spectroscopy (VTC-XES) at the Pt L_3_-edge. VTC-XES measurements reveal that the Pt d-band centroid shifts away from the Fermi level upon dilution, accompanied by concentration-dependent Pt d-band width. The competition between the strain effect and ligand effect is observed experimentally for the first time. It is found that the d-band widths in Pt_3_Ni and PtNi are broader than that of Pt metal due to compressive strain which overcompensates the effect of dilution, while it is narrower in PtNi_3_ where the ligand effect dominates. VTC-XES is demonstrated to be a powerful tool to study the Pt d-band contribution to the valence band of Pt-based bimetallic. The implication for the enhanced activity of Pt-Ni catalysts in oxygen reduction reaction is discussed.

## Introduction

Pt-based alloys have been studied as one of the most promising catalysts for oxygen reduction reaction (ORR) in proton exchange membrane fuel cells (PEMFCs)^1,2,3^. Compared with pure Pt, which is a good ORR catalyst, the introduction of a transition metal (Ni, Co, etc.) provides tunability for the Pt lattice constant (strain effect due to size mismatch in a fcc random alloy or compound for example) and valence band (redistribution of Pt 5d states) in the form of alloys (intermetallic interaction) or core–shell structures (strain effect due to size mismatch of the core and the shell). In principle, the change of chemical environment (coordination species, interatomic distance, etc.) of Pt results in the redistribution of 5d densities of states (centroid shift and band width change), which directly determines the binding strength of the adsorbate species through bonding and anti-bonding interactions with the catalyst. The configuration of the Pt d-band plays an important role in determining the densities of states in the vicinity of the Fermi level, both occupied and unoccupied, hence its catalytic activity, which normally exhibits a volcano curve behavior towards the adsorbate binding strength^4^. The tuning of the Pt valence band to optimize the reactivity and selectivity is the most effective means towards Pt-based catalysts engineering. The distribution of the Pt 5d character in the valence band of an alloy, however, cannot be easily determined by conventional laboratory techniques. Ultraviolet photoelectron spectroscopy (UPS), for example, can detect the total valence band of Pt-Ni alloys; it has little elemental sensitivity to distinguish between the Pt and Ni character in the valence band and is surface sensitive.

In the past decade, valence-to-core X-ray emission spectroscopy (VTC-XES) has evolved to become a synchrotron technique for valence band detection with elemental specificity, as sophisticated high energy resolution X-ray crystal analyzers emerged. The elemental specificity is achieved by tuning the excitation energy across the absorption edges (core levels) of interest and tracking the inelastically scattered X-rays (just below the edge) and the fluorescence X-rays (when the core hole is switched on) using high energy resolution X-ray optics and area sensitive detectors. This is the so-called high energy resolution fluorescence detected (HERFD) setup, which uses either a cylindrically bent crystal with a dispersive geometry (von Hamos design)^5,6,7,8^ or a spherically bent crystal analyzer (SBCA) in a Rowland circle^9,10^. This development was propelled by the interest in resonant inelastic X-ray scattering (RIXS). RIXS is a concerted phenomenon of absorption and emission, has maximum intensity at threshold and can circumvent core–hole lifetime broadening; all these however require high energy resolution detection to be observed experimentally in the hard X-ray regime. As a result of the evolution of the bright and tunable incident X-ray and high energy resolution detection, the valence band of heavy metal elements can now be experimentally determined from VTC-XES. This has been recently demonstrated by studies of metal–ligand interactions for Ru complexes by Biasin et al.^11^, Levin et al.^12^, and Mn complexes by Hall et al.^13^. In this study, VTC-XES is conducted at the Pt L_3_-edge with great signal/noise (compared with 1s excitation on transition metals) due to high energy resolution and the dipole-allowed 5d_5/2,3/2_→2p_3/2_ transition.

**Figure 1:**
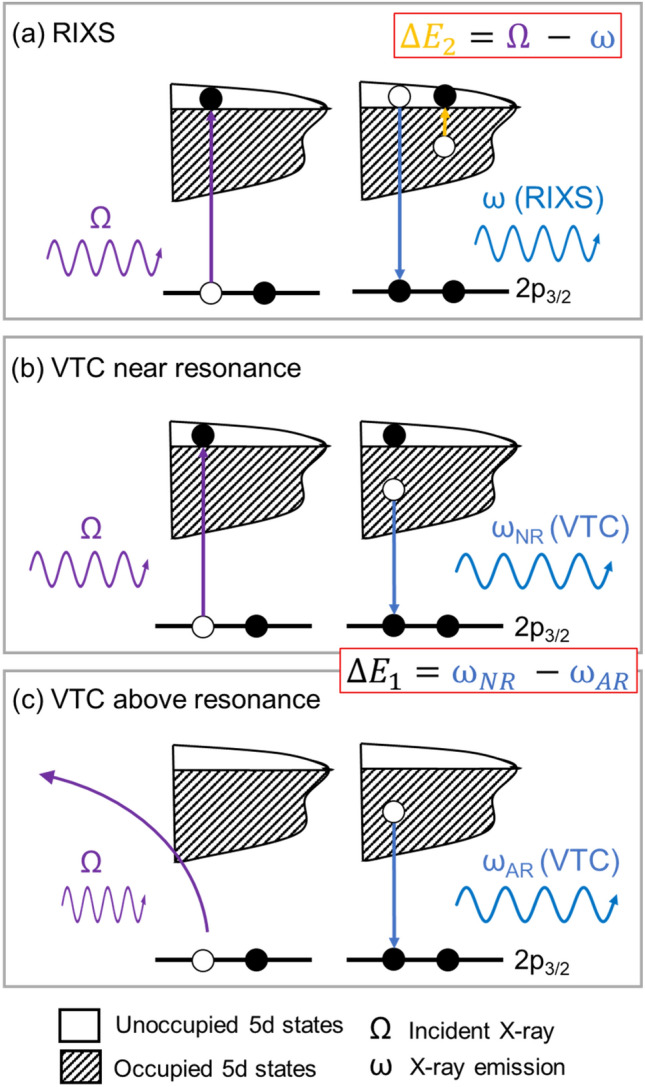
X-ray emission process associated with the Pt d band including the (**a**) RIXS, the decay of the intermediate state and the shakeup process (valence excitation) are a concerted process; (**b**) VTC near Pt L_3_-edge; and (**c**) VTC above Pt L_3_-edge. ω_NR_ and ω_AR_ represent the VTC X-ray emission with excitation near resonance and above resonance, respectively as defined in the text.

The X-ray emission process with the excitation energy scanned across the Pt L_3_-edge is illustrated in Fig. 1 in three energy regions: (a) RIXS, ω resulting from an intermediate state and final state differentiated by the excitation of a valence electron into the conduction band via energy loss (ΔE_2_ = Ω—ω), by the incident X-ray, Ω; the process is enhanced by the proximity of the Pt 2p_3/2_ to 5d_5/2,3/2_ dipole transition; (b) VTC above threshold (E_0,_ the point of inflection of the rising absorption edge) and near resonance (within the WL); and (c) VTC above resonance (non-resonant XES). Figure 1a shows the RIXS region where the emission energy exhibits a dispersion. The energy loss ΔE_2_ (Pt 5d band maximum) is a constant as the excitation energy (Ω) scans across the adsorption edge until it reaches the threshold then the core hole is turned on and resonant X-ray emission (fluorescence) takes place. In Fig. 1b where the excitation energy is above the threshold but not high enough to excite the core electron into the continuum, the VTC emission, as the name suggests, originates from a Pt 5d electron in the valence band combining with a 2p_3/2_ core hole via dipole transition, emitting a fluorescence photon. In Fig. 1c, at a few eV above the resonance (whiteline maximum), the excited core electron has enough kinetic energy to escape the Pt atom, and the VTC emission (ω_AR_) is still tracking the Pt 5d in the valence band but losing the advantage of enhanced intensity and core–hole lifetime broadening suppression. The absorption and emission processes are no longer concerted.

In this study, the VTC emission of Pt-Ni alloys is studied with HERFD detection at the Sector 20-ID beamline of the Advanced Photon Source (APS). A Si (311) SBCA using the (933) reflection (~ 1 eV resolution) is coupled to a PILATUS 100 K-S 2D detector (DECTRIS Ltd., Switzerland) in a Rowland Circle, allowing for the simultaneous collection of the X-ray emission from the entire Pt 5d band. The incident (excitation) X-rays from the Si (111) monochromators provides an energy resolution of 1.4 × 10^–4^ near the Pt L_3_-edge. Pt-Ni alloys including Pt_3_Ni, PtNi, and PtNi_3_ were synthesized by vacuum arc-melt method^14^. The crystal structure, local structure, and electronic structure have been reported using X-ray diffraction, normal mode X-ray absorption spectroscopy, ultraviolet photoelectron spectroscopy (UPS), and density functional theory calculation (DFT). Charge transfer from Ni to Pt (by filling the Pt 5d holes) upon alloying has been established^14,15^. Experiment with elemental sensitivity, however, is still in need to provide direct evidence of how the Pt valence band, especially the Pt 5d states, changes upon alloying, which in turn determines its catalytic behavior. Herein, with the state-of-the-art VTC-XES, we report the observation of chemical shifts and both broadening and narrowing of the Pt 5d band in the Pt-Ni alloy valence band due to competing strain and ligand effects.

## Results and discussion

**Figure 2:**
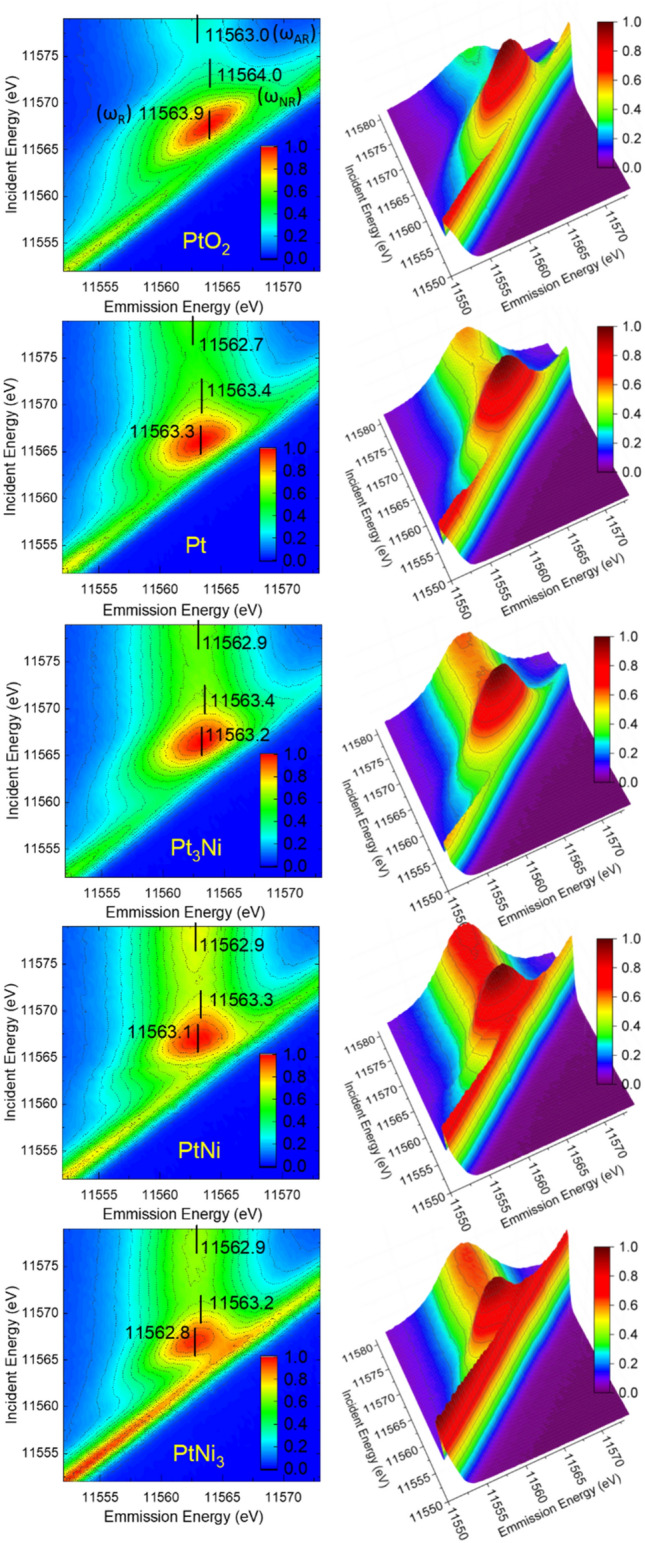
XES near the Pt L_3_-edge presented in two-dimensional and three-dimensional plots, showing the normalized intensity of RIXS and ES. The vertical bar on the left panel tracks the position of the emission in different energy region of excitation at (ω_R_), near (ω_NR_) and above (ω_AR_) the resonance. The diagonal peak in the right panel displays the relative intensity of the elastic peaks.

Figure 2 displays the 2D and 3D plots of excitation vs emission X-ray energies across the Pt L_3_-edge with the intensity color coded. In the emission panel, the energy of the emitted X-ray (collected by the area sensitive detector) is very close to the excitation energy which will also appear in the spectrum as an elastically scattered (ES) peak, as shown in the right column of Fig. 2. A clear trend of increasing intensity of the ES (diagonal) is observed as Pt becomes more diluted in Ni. This is because Ni mainly contributes to elastic X-ray scattering instead of absorption at X-ray energies near the Pt L_3_-edge. In PtNi_3_, the most dilute case, the ES signal is strong enough to partially overlap with the VTC emission, while it is least intense in Pt_3_Ni. Note that the Kapton tape as the sealing material for the Pt foil and alloy samples also contributes to the ES peak. Also note that the quasi-Pt 5d_3/2_ and 5d_5/2_ band features are not resolved in the VTC emission spectra because the energy separation of the 5d spin–orbit derived features in the valence band is smaller than the energy resolution.

In the left panel of Fig. 2, ω_R_ is the emission energy from excitation at whiteline (WL) maximum, ω_NR_ is from excitation near the resonance within the WL envelope and ω_AR_ is beyond the WL_._ They are marked with vertical bars and tabulated in Table 1. The WL exhibits a trend consistent with Ni → Pt charge transfer upon alloying. The VTC emission energies near the resonance ω_NR_ and above resonance ω_AR_ result in the energy difference ΔE_1_ = ω_NR_ – ω_AR_ (1.0, 0.7, 0.5, 0.4, and 0.3 eV for PtO_2_, Pt, Pt_3_Ni, PtNi, and PtNi_3_, respectively), also shown in Table 1. This difference is always positive as expected from the energy difference between the adiabatic and the sudden regime in core hole screening; that is that the addition of the excited electron in the unoccupied Pt 5d states above the Fermi level leads to a better screened core hole and a larger X-ray emission energy. Recall that both ω_NR_ and ω_AR_ are the X-ray emission from the recombination of a valence electron of Pt 5d character and a 2p_3/2_ core hole. ΔE_1_ hence arises from the existence of the excited electron in the vicinity of the Pt atom. There is a clear trend in the chemical systematic in that the more Pt 5d holes there are, the larger the ΔE_1_. This is reasonable since more available 5d holes will lead to poorer screening, hence a larger chemical shift.

**Table 1:** Summary of emission energies ω_NR_, ω_AR_, ω_R_, energy difference ΔE_1_, energy transfer ΔE_2_, and FWHM of the samples. The detailed analyses are shown in the SI.

Sample	ω_NR_ (± 0.1 eV)	ω_AR_ (± 0.1 eV)	ω_R_ (± 0.1 eV)	ΔE_1_ (eV)	ΔE_2_ (eV)	FWHM^a^ (eV)	FWHM ^b^ (eV)	FWHM^c^ (eV)
PtO_2_	11,564.0	11,563.0	11,563.9	1.0 ± 0.1	3.3 ± 0.1	5.8 ± 0.2	6.1 ± 0.1	8.1 ± 0.2
Pt	11,563.4	11,562.7	11,563.3	0.7 ± 0.1	2.5 ± 0.1	6.5 ± 0.1	7.0 ± 0.1	7.9 ± 0.2
Pt_3_Ni	11,563.4	11,562.9	11,563.2	0.5 ± 0.1	2.8 ± 0.1	6.8 ± 0.1	7.0 ± 0.1	7.9 ± 0.1
PtNi	11,563.3	11,562.9	11,563.1	0.4 ± 0.1	3.0 ± 0.1	6.6 ± 0.1	7.0 ± 0.1	7.9 ± 0.1
PtNi_3_	11,563.2	11,562.9	11,562.8	0.3 ± 0.1	3.1 ± 0.1	6.3 ± 0.1	6.8 ± 0.1	7.8 ± 0.1

The step size of the raster scan is 0.5 eV (for both the excitation and emission energies). The error analyses are based on the smoothed spectra, with uncertainty of ~ 0.1 eV in emission energy.

^a^Excitation energies are below resonance, at 11,560.8–11,562.8 eV. Averaged values are displayed.

^b^Excitation energies are at resonance.

^c^Excitation energies are above resonance, at 11,578.5 ~ 11,580.5 eV. Averaged values are displayed.

We also note that the emission centroid at the resonance ω_R_ (11,563.9, 11,563.3, 11,563.2, 11,563.1, and 11,562.8 eV for PtO_2_, Pt, Pt_3_Ni, PtNi, and PtNi_3_, respectively, see Table 1) is slightly different from ω_NR_. This is because of the interference of the RIXS signal, which spans along the energy transfer direction (diagonal in Fig. 2) and associated chemical effect (5d hole population), resulting in the elongation and asymmetry of the emission pattern near the resonance. The elongation effect is most evident in PtO_2_ where Pt 5d electrons are partially transferred to oxygen. Correspondingly, more 5d holes are available to accommodate the intermediate states in the RIXS process, hence the XES pattern is elongated by the RIXS to the highest extent (see Figure S1). In comparison, Pt attracts electrons from Ni in the Pt-Ni alloys to fill the d band, thus the elongation becomes less severe than in pure Pt.

**Figure 3:**
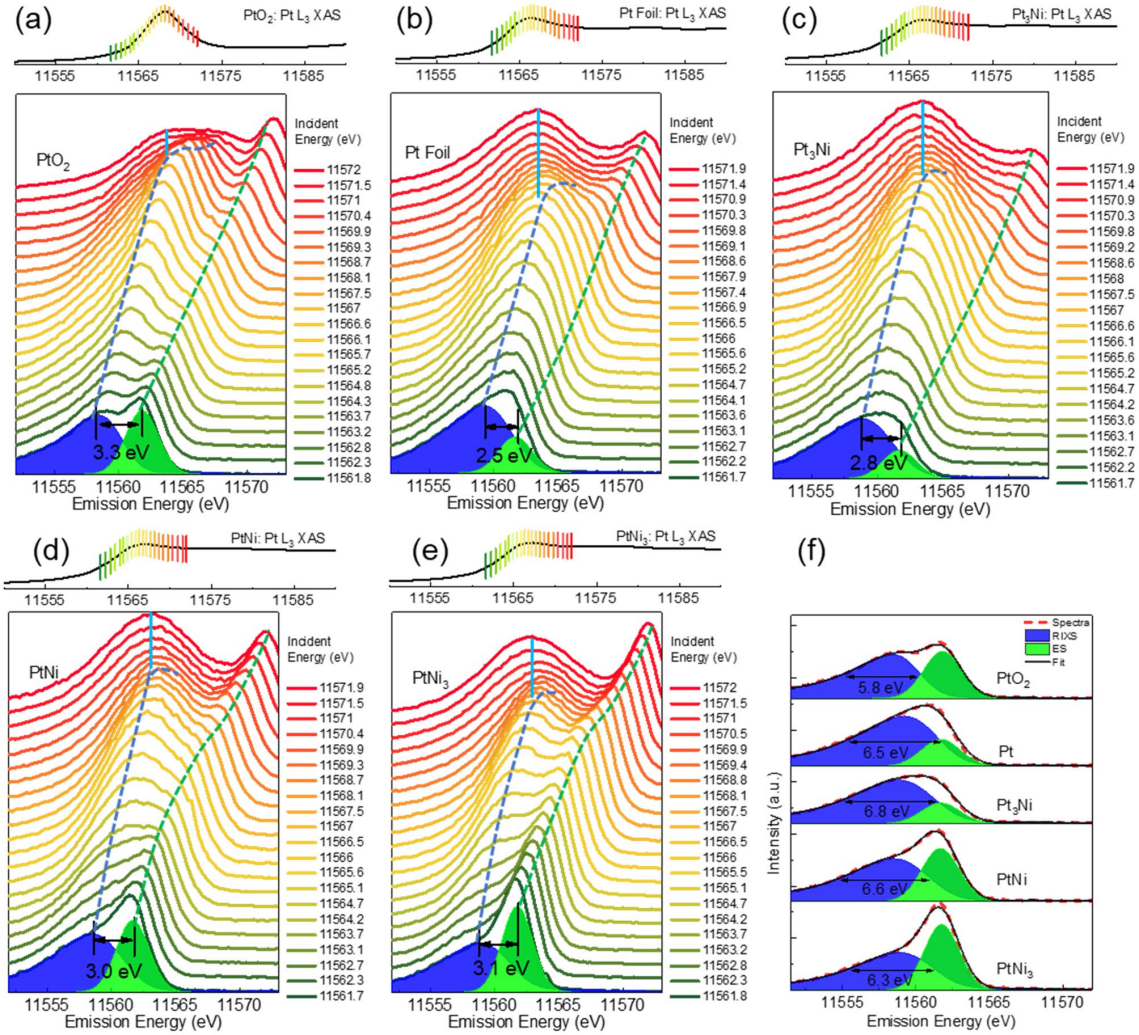
X-ray emission spectra from (**a**) PtO_2_, (**b**) Pt foil, and (**c**–**e**) Pt-Ni alloys collected across the Pt L_3_-edge with the corresponding XAS collected in normal FY mode. The energy difference (ΔE_2_) between the RIXS peak (blue dash line) and ES peak (green dash line) below WL is labeled for each sample, with the FWHM of the inelastic peaks summarized in (**f**). ΔE_2_ and FWHM are averaged numbers extracted from five spectra excited at 11,560.8–11,562.8 eV. The VTC X-ray emission ω_NR_ is labeled in light blue straight line. The ES peak is fitted using a Gaussian peak with the half width obtained from the ES signal at the excitation energy of 11,555 eV. The detailed analysis is described in the SI.

To obtain the precise Pt 5d distribution in the valence band, the XES spectra excited across the Pt L_3_-edge WL for each sample are extracted (Fig. 3). Figure 3a–e show the VTC-XES for the five compounds as the excitation energy scans across the WL. One can clearly see the dispersion of the ES (dash green curve), the RIXS (dash blue curve) and the emergence of the fluorescence (solid blue line). Five XES recorded at the excitation energies of 11,560.8–11,562.8 eV are fitted with a Gaussian profile (FWHM = 3.0, 3.1, 3.1, 3.0, and 2.8 eV for PtO2, Pt, Pt_3_Ni, PtNi, and PtNi_3_, respectively, determined from the ES signal at the excitation energy of ~ 11,555 eV for each sample) and the RIXS signal (Pt d band). The results are shown in Table S2, Figure S4, S5, and summarized in Fig. 3f where the green peak is the elastic peak (~ 11,562 eV), and the blue band is the Pt 5d component of the VB of the alloy defined by a peak maximum and a band width. To check the validity of these values, we consider the ES width and the Pt d band in quadrature ($6.5=\sqrt{{\Delta }_{5d}^{2}+{\Delta }_{\mathrm{E}\mathrm{S}}^{2}}$ ), The observed 6.5 eV width yields a Pt 5d band width Δ_5d_ of 5.7 eV, which is consistent with that observed in UPS and predicted by theory^14^.

The energy transfer (3.3, 2.5, 2.8, 3.0, and 3.1 eV for PtO_2_, Pt, Pt_3_Ni, PtNi, and PtNi_3_, respectively), ΔE_2_ = Ω – ω, represents the energy loss to the excitation of the Pt 5d valence electrons to the narrow unoccupied d states just above the Fermi level; in other words, it is the binding energy of the centroid of the Pt 5d states below the Fermi level. For each sample, ΔE_2_ is a constant. In the case of PtO_2_, a relatively large ΔE_2_ of 3.3 eV is expected because of the bandgap (> 1.0 eV)^16,17,18^. In the cases of Pt metal and alloys, ΔE_2_ shifts away from the Fermi level as Pt becomes more diluted in Ni. The same trend is observed in the DFT calculation results of these alloys in our previous study, attributing to the redistribution of Pt 5d states upon alloying with Ni^14^, and also in good agreement with the theoretical study by Matanovic et al., where the d-band centers of Pt were calculated to be − 2.01, − 2.17, − 2.31 and − 2.56 eV for Pt, Pt_3_Ni, PtNi, and PtNi_3_, respectively^19^. Similar behavior is also observed in the Au d band in Au-Cu^20^ and Au-Pt alloys^21^. The most exciting observation is in the full width at half maximum (FWHM): 5.8, 6.5, 6.8, 6.6, and 6.3 eV for PtO_2_, Pt, Pt_3_Ni, PtNi, and PtNi_3_, respectively. The results are summarized in Table 1. The difference is significantly above uncertainty. Note that these excitation energies were selected because they are in the RIXS region with an enhanced cross-section and suppressed core–hole lifetime broadening, hence it will provide higher chemical sensitivity compared with the VTC emission at and above the WL. The reason for this is further explained in Figure S1 which shows that the VTC becomes broader as the excitation energy moves across the threshold. For PtO_2_, a smaller width is observed because it is Pt (IV) with depletion of Pt 5d electrons compared to Pt metal, and there is little Pt–Pt interaction in the nearest neighbors hence a narrow Pt d band.

We now focus on the difference in the band width between Pt metal and the alloys. We note that Pt_3_Ni (6.8 eV) and PtNi (6.6 eV) show larger band widths than that of Pt (6.5 eV), while that of PtNi_3_ (6.3 eV) is narrower. At excitation energies near, at, and above the resonance, a similar trend is observed that the band widths of Pt_3_Ni and PtNi are comparable to that of Pt, while PtNi_3_ shows the narrowest, as shown in Figure S2, S3 and Table S1. Note that near the resonance, the RIXS signal and VTC emission can partly overlap and broaden the overall emission line, as indicated in Figure S1. At above resonance, the variance of the band widths is mitigated because of the broadened feature although the interference from ES is excluded. Herein, PtO_2_ at above resonance shows an exceptionally wide emission band because of the broadening of the asymmetric feature (see Figure S3). Among the metallic Pt, the anomaly of band width widening upon dilution, though has never been observed before, can be rationalized. Let us return to the Pt catalysts engineering strategy discussed in the introduction. We consider two competing effects: (1) the compressive strain effect arising from the smaller size of Ni, resulting in shorter Pt–Pt bond in the compressed alloy lattice compared with Pt metal; compression of the Pt–Pt interatomic distance will lead to a wider band (the Pt–Pt interatomic distance is 0.041 ± 0.005 Å shorter in Pt_3_Ni, and 0.081 ± 0.005 Å shorter in PtNi compared to that in Pt metal)^14^; and (2) the ligand (Ni) effect due to the presence of Ni as a nearest neighbor of Pt reduces the Pt–Pt coordination number and is expected to result in a narrower band. The conventional wisdom is that dilution of a transition metal in a metallic host will usually narrow the d band, and the above-mentioned ligand effect often prevails. However, the strain effect, which is often seen in core–shell nano-systems where Pt is the shell and a substrate with atoms of a smaller size is the core leading to the compression of the shell and shorter Pt–Pt interatomic distance, should induce band broadening due to repulsion. The competing strain and ligand effect have never been observed until now because it requires a bright excitation source and a crystal analyzer with high energy resolution for both the incident and the emitted X-rays. Compared with the width from the Pt foil (5.7 eV), Δ_5d_, from quadrature analysis, Pt_3_Ni and PtNi show larger d band width (6.1 and 5.9 eV, respectively), indicating that the strain effect dominates in the Pt_3_Ni and PtNi. This phenomenon is supported by the theoretical study of the Pt(111) surface on subsurface Ni^22^. It should be noted that the trend of Pt 5d band width is slightly different from UPS results^14,23^ which cannot separate the Pt d band from Ni d band in the alloys. The overall trend from this study as well as the previous results indicate that the Pt d-band center shifts away from the Fermi level regardless of the band width upon alloying with Ni. These changes are expected to have an impact on the chemical activity when Pt-Ni alloys are used as the catalysts. For example, in ORR where the binding strength of oxygen species on the Pt surface is slightly stronger relative to the optimal condition, the above discussed Pt-Ni alloys are supposed to weaken the binding strength compared with Pt. On one hand, a higher filled (electrons are transferred from Ni to Pt) Pt 5d states will result in a higher filled Pt-O anti-bonding orbitals formed by Pt 5d and O 2p orbitals, which weakens the binding strength upon surface adsorption. On the other hand, the shifting of the Pt valence states to higher binding energy (further away from the Fermi level) is also expected to increase the filling of the Pt-O anti-bonding orbitals. This mechanism may be responsible for the enhanced specific activity in ORR for Pt-Ni alloys with various compositions^3,24,25,26,27^. Meanwhile, it is worth noting that the oxygen binding strength on Pt in ORR is not the weaker the better. In the case of weak binding, the adsorption of oxygen as well as the subsequent protonation of oxygen will become the limiting step of ORR and hence decrease the catalytic activity^28^. Therefore, it is important to accurately characterize the Pt valence states when establishing design strategies of future catalysts.

## Conclusions

VTC-XES has been conducted with excitation near the Pt L_3_-edge with precision to provide direct experimental evidence for the behavior of the Pt 5d distribution in the valence band upon alloying with Ni. The Pt 5d band centroid is found to shift away from the Fermi level upon dilution in Ni. The variation of the Pt 5d band in the Pt-Ni alloys compared with that of pure Pt is found to broaden in Pt_3_Ni and PtNi, and then becomes narrower in PtNi_3_. The result is attributed to the competition between the strain effect and the ligand effect; that is that in Pt_3_Ni and PtNi where the strain effect (shorter Pt–Pt bond length) has overcompensated the dilution effect (reduction in coordination number), yielding a wider Pt 5d band in the alloy compared with the pure metal. The chemical shift of the VTC as the excitation energy scans across the resonance is found to be associated with the 5d hole counts, which accounts for the improved activity in Pt-Ni catalysts. The VTC emission panel including the RIXS provides rich information for the Pt 5d characters in the valence band of the Pt-Ni alloys. From these results, VTC-XES in the hard X-ray range has been demonstrated to be a powerful technique to study the d character of valence band of d band metals and alloys. This technique is especially useful for complicated systems such as Pt based bimetallic core–shell nano catalysts, and in situ/operando studies where the valence band can be altered by adsorbates, morphology, heterostructures, etc.

## Supplementary Information

Supplementary Information.
