# Supplementary material for: Strain and ligand effects in Pt-Ni alloys studied by valence-to-core X-ray emission spectroscopy
Source: Sci Rep. 2021 Jul 1;11:13698. doi: 10.1038/s41598-021-93068-0 (PMC8249455; doi:10.1038/s41598-021-93068-0)
Supplement: Supplementary file 1 — Supplementary Information. [file 41598_2021_93068_MOESM1_ESM.docx]

Strain and ligand effects in Pt-Ni alloys studied by valence-to-core X-ray emission spectroscopy

Supplementary Information

Jiatang Chen^1^, Y. Zou Finfrock^2,3^, Zhiqiang Wang^1^, Tsun-Kong Sham^1,*^

^1^ Department of Chemistry, University of Western Ontario, London, Ontario N6A 5B7, Canada

^2^ CLS@APS Sector 20, Advanced Photon Source, Argonne National Laboratory, Lemont, IL 60439, USA

^3^ Science Division, Canadian Light Source Inc., Saskatoon, Saskatchewan S7N 2V3, Canada

**Elongation of the XES pattern**

For the elongation of the XES pattern, the graph below provides an illustration. The increasing unoccupied 5d states (PtO_2_ > Pt > PtNi_3_) leads to more intermediate states for RIXS, and thus stronger RIXS signal in the diagonal (elongation).


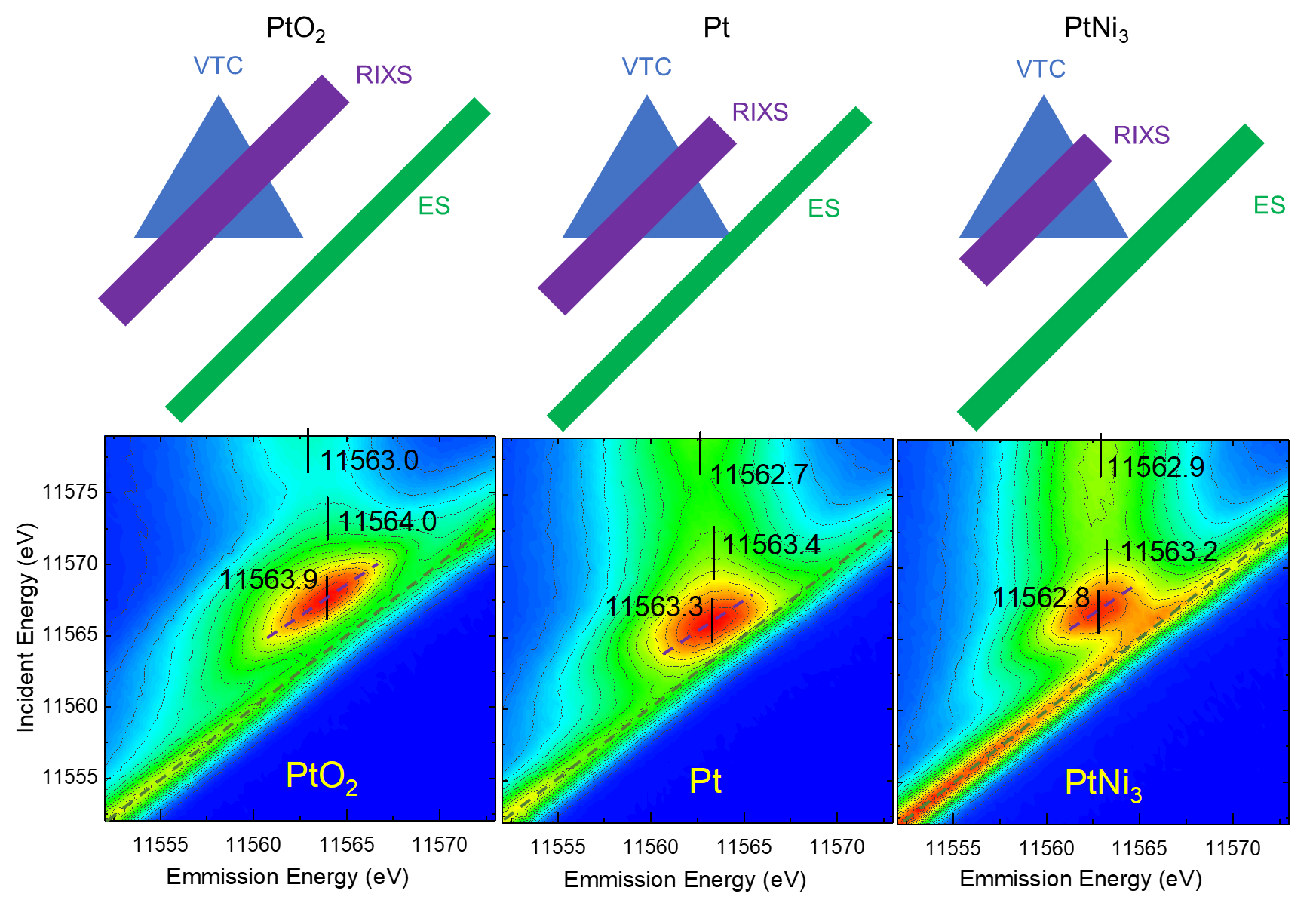


Figure S1. The subtle behavior of RIXS and the elastic scattering (ES) peak along the diagonal of the pseudo 2D plot as a function of Pt 5d hole population with the trend: PtO_2_ > Pt > PtNi_3._ The nominal 5d hole in PtO_2_ is greater than 2, the 5d hole is 1.34 and 0.76 ^1^, for Pt and PtNi_3_, respectively. The lines in the plots are to guide the eye. The illustration above the 2D plot is a simplified version of the trend. The ES is more intense in the dilute sample PtNi_3_ as noted in the text.

**XES at excitation energy above the WL resonance**

At excitation above the WL, the fluorescence channel is fully turned on and the RIXS lingers but decays rapidly and the excitation and fluorescence are no longer concerted. This will lead to core-hole life broadening. From the results below in Figure S2, we can see the trend of broadening from excitation with photon energy just below, at, and above resonance (WL maximum). Thus, the selection of excitation below instead of above the threshold, is an optimum choice for VTC measurements.

**
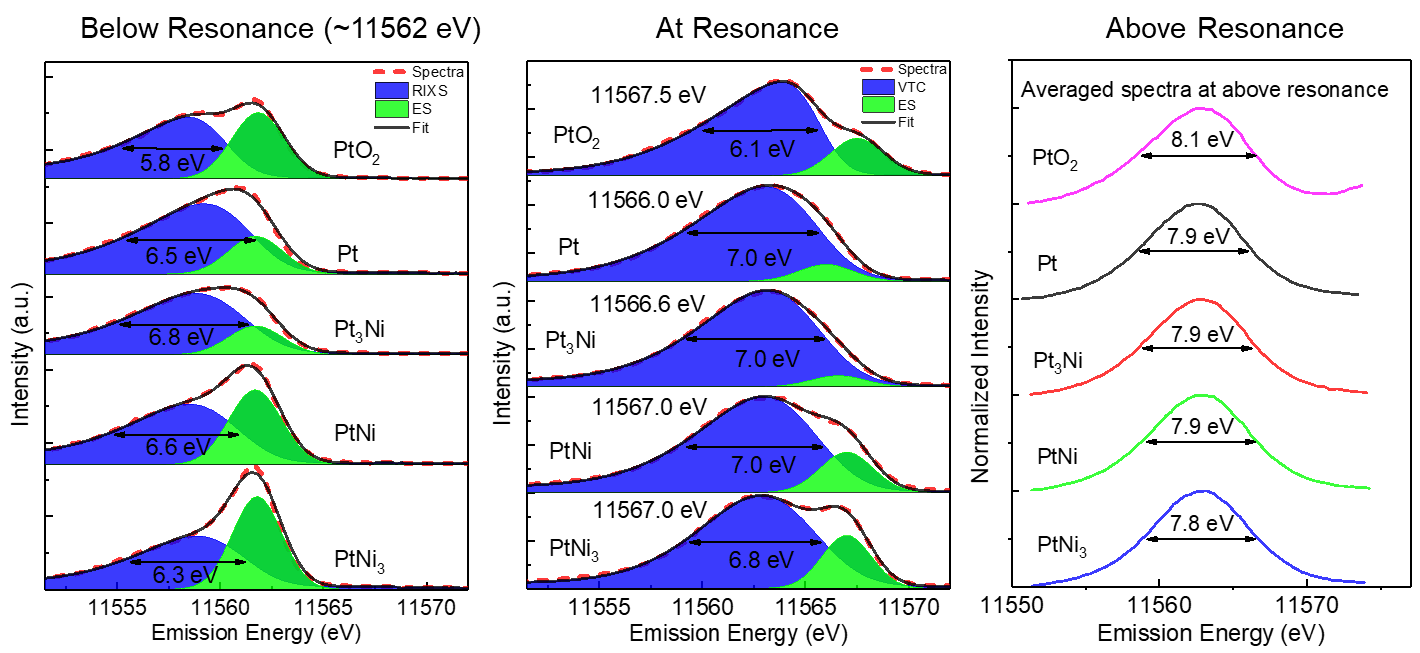
**

Figure S2. Variation of the Pt 5d band width as a function of excitation energy in the RIXS region below WL resonance (left panel, excitation: ~11562 eV) at the WL peak (mid panel, the excitation energy for each sample is labeled) and above (right panel, averaged spectra for each sample at the excitation energies of 11578.5~11580.5 eV). The observed widths are given in the plots. A systematic broadening is observed as expected from the modified Kramers-Kronig equation.

**Detailed analysis of bandwidths**

1. **Statistical analysis of the bandwidths above resonance.**

Five spectra extracted at the excitation energies from 11578.5 to 11580.5 eV are used for the bandwidth (FWHM) analyses. The raw and averaged spectra are shown in Figure S3. The summarized bandwidths are shown in Table S1.

Table S1. Standard error analysis of extracted bandwidth at above resonance. All numbers are in the unit of eV. The final error is obtained from the coupling of standard error with the measuring uncertainty of each spectrum, i.e. 0.1 eV for each energy point and 0.14 eV for measuring a distance in the smooth spectra.

| Sample | Measured bandwidths (eV) at above resonance energies (eV) | | | | | Averaged  bandwidth | Standard error | Final error |
| --- | --- | --- | --- | --- | --- | --- | --- | --- |
|  | ~11578.5 | ~11579.0 | ~11579.5 | ~11580.0 | ~11580.5 |  |  |  |
| PtO2 | 8.2 | 8.4 | 8.0 | 7.9 | 8.1 | 8.1 | 0.08 | 0.2 |
| Pt | 8.1 | 8.0 | 7.9 | 7.8 | 7.7 | 7.9 | 0.07 | 0.2 |
| Pt_3_Ni | 8.0 | 7.8 | 7.9 | 7.8 | 8.0 | 7.9 | 0.05 | 0.1 |
| PtNi | 7.9 | 7.9 | 7.9 | 8.0 | 7.9 | 7.9 | 0.02 | 0.1 |
| PtNi_3_ | 7.8 | 7.8 | 7.6 | 7.9 | 7.8 | 7.8 | 0.04 | 0.1 |

1. **Statistical analysis of the bandwidths and energy transfer below resonance.**

The width of the fitted RIXS or VTC emission signal is sensitive to the width of elastic scattering signal (ES). Meanwhile, the elastic signal for each sample can vary (in a small range) among different samples because of the different elemental composition and Kapton tape (sealing material). It is therefore important to extract the correct ES individually for each sample. The following procedure is used in the fitting process:

1. The ES for each sample is extracted by fitting the XES at the incident energy of ~11555 eV (below Pt L_3_-edge), as shown in Figure S4.
2. The ES (Gaussian peaks with the widths of 3.0, 3.1, 3.1, 3.0, and 2.8 eV for PtO_2_, Pt, Pt_3_Ni, PtNi, and PtNi_3_, respectively) is used to fit the 5 XES at the incident energies of 11560.8~11562.8 eV (below resonance) to extract the RIXS. A Shirley background is used. The energy position and Gaussian width of ES is fixed. The intensity of ES, the position and intensity of an asymmetric peak are variables to obtain the best fit. The obtained asymmetric peak together with the Shirley background are considered as the RIXS signal (equals to the the total signal subtracting the ES). The FWHM of the RIXS signal is extracted as the bandwidth of RIXS. The distance between the RIXS maximum and the ES peak position is measured as the energy transfer ΔE_2_ in the manuscript. The fitting results are shown in Figure S5.
3. The fitted FWHM and ΔE_2_ are statistically analyzed respectively for each sample to obtain the average values and standard errors, as summarized in Table S2.
4. The final error is calculated as $\sqrt{\left( standard error \right)^{2}+{(measuring error)}^{2}}$, where the measuring error is considered as $\sqrt{\left( 0.1 \right)^{2}+{(0.1)}^{2}}=0.14 eV$ for FWHM and ΔE_2_.

Table S2. Statistical analysis of the extracted FWHM of RIXS and energy transfer ΔE_2_ below resonance. The final error is obtained from the coupling of standard error with the measuring uncertainty of each spectrum, i.e. 0.1 eV for each energy point and 0.14 eV for measuring a distance in the smooth spectra.

| Sample | PtO_2_ (eV) | | Pt (eV) | | Pt_3_Ni (eV) | | PtNi (eV) | | PtNi_3_ (eV) | |
| --- | --- | --- | --- | --- | --- | --- | --- | --- | --- | --- |
| Excitation Energy (eV) | ΔE_2_ | FWHM | ΔE_2_ | FWHM | ΔE_2_ | FWHM | ΔE_2_ | FWHM | ΔE_2_ | FWHM |
| ~11560.8 | 3.3 | 5.6 | 2.5 | 6.4 | 2.8 | 6.8 | 3.0 | 6.6 | 3.0 | 6.3 |
| ~11561.3 | 3.3 | 5.8 | 2.4 | 6.6 | 2.9 | 6.8 | 3.1 | 6.6 | 3.0 | 6.3 |
| ~11561.8 | 3.3 | 5.7 | 2.5 | 6.6 | 2.9 | 6.8 | 3.0 | 6.6 | 3.0 | 6.3 |
| ~11562.3 | 3.4 | 5.9 | 2.5 | 6.4 | 2.8 | 6.8 | 3.0 | 6.7 | 3.1 | 6.3 |
| ~11562.8 | 3.4 | 5.9 | 2.5 | 6.5 | 2.8 | 6.8 | 3.1 | 6.6 | 3.2 | 6.4 |
| Average | 3.3 | 5.8 | 2.5 | 6.5 | 2.8 | 6.8 | 3.0 | 6.6 | 3.1 | 6.3 |
| Standard error | 0.02 | 0.06 | 0.02 | 0.04 | 0.02 | 0.00 | 0.02 | 0.02 | 0.04 | 0.02 |
| Final error | 0.1 | 0.2 | 0.1 | 0.1 | 0.1 | 0.1 | 0.1 | 0.1 | 0.1 | 0.1 |

1. **Bandwidth at resonance**

The bandwidth at resonance for each sample was extracted from only one spectrum (closest to the maximum). Because the excitation energy of resonance maximum is also the crossing point of RIXS and VTC signal, a slight shift (in excitation energy) from the crossing point will lead to mismatch of two signals and thus broadening of the spectrum (see Figure S1). Meanwhile, the scanning step of excitation energy was set to be 0.5 eV, which will inevitably introduce this broadening when extracting the XES spectrum at resonance (the excitation energy is almost impossible to coincide with the crossing point). The spectra at resonance maximum are therefore not considered as important as those below resonance in the bandwidth analysis.

**
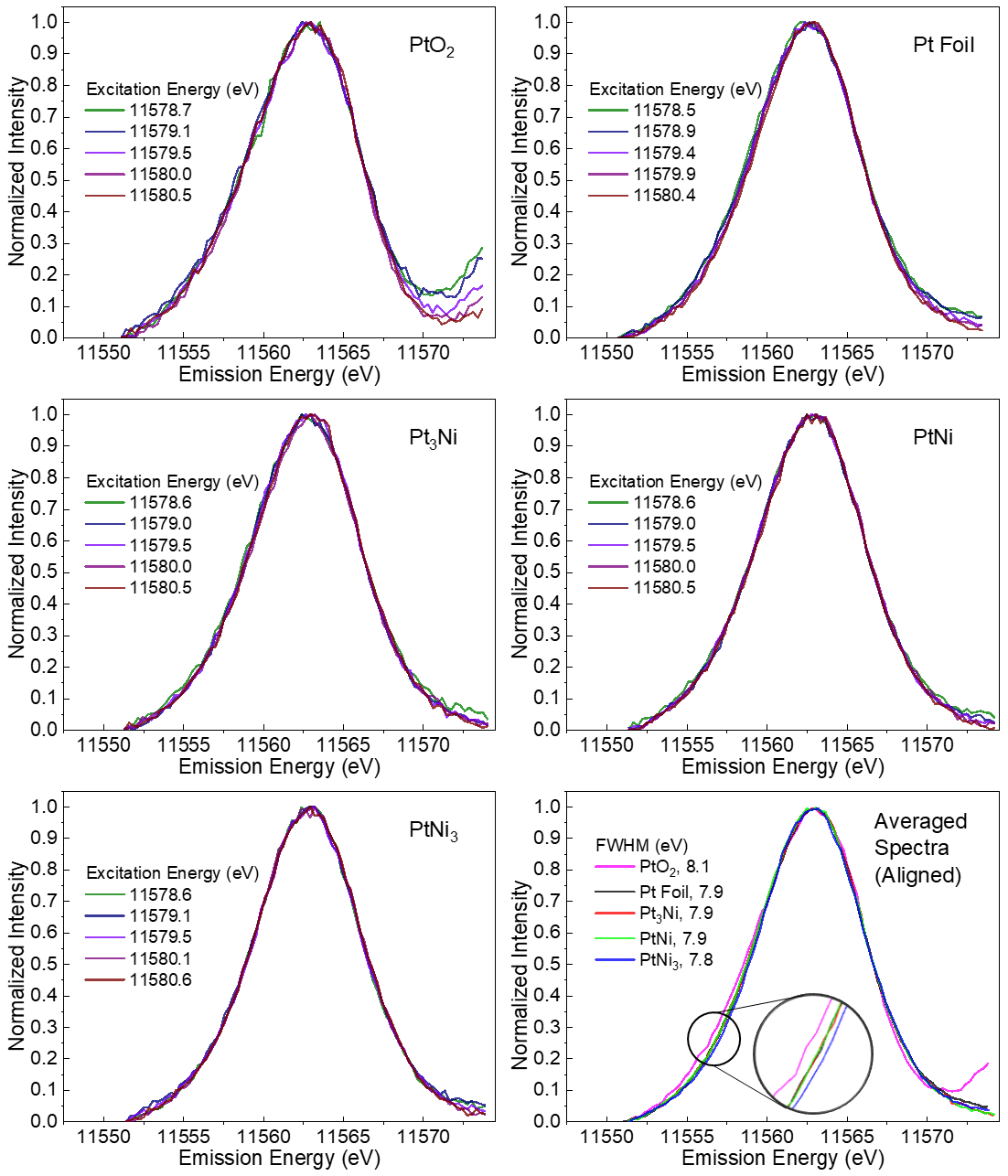
**

Figure S3. The raw spectra and averaged spectrum for each sample at the excitation energy of 11578.5 ~ 11580.6 eV (above resonance).The averaged spectra are aligned at the half maximum point at the higher emission energy side. The inset of shows the statistically narrowed Pt 5d bandwidth of PtNi_3_ (blue) compared with other samples. The anomaly large bandwidth of PtO_2_ (purple) may be caused by the broadening (at above resonance) of the more asymmetric band shape (due to the loss of 5d electrons to oxygen atoms).


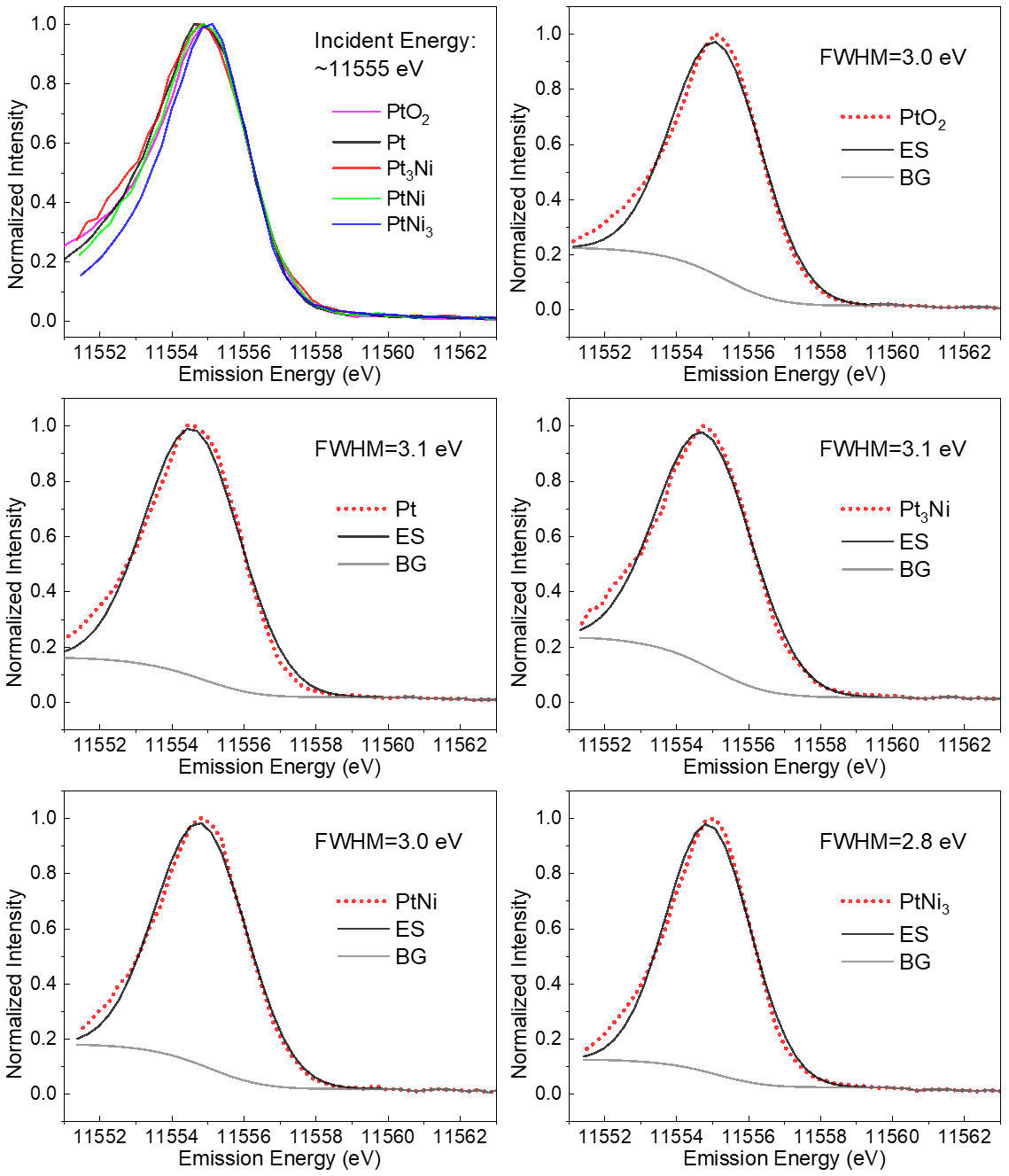


Figure S4. Extraction of the width (FWHM) of the elastic scattering signal for each sample by fitting the XES at ~11555 eV with a Gaussian peak (ES) and a Shirley background (BG). The raw spectra are shown in the upper-left graph and as red dots in each fitting graph.


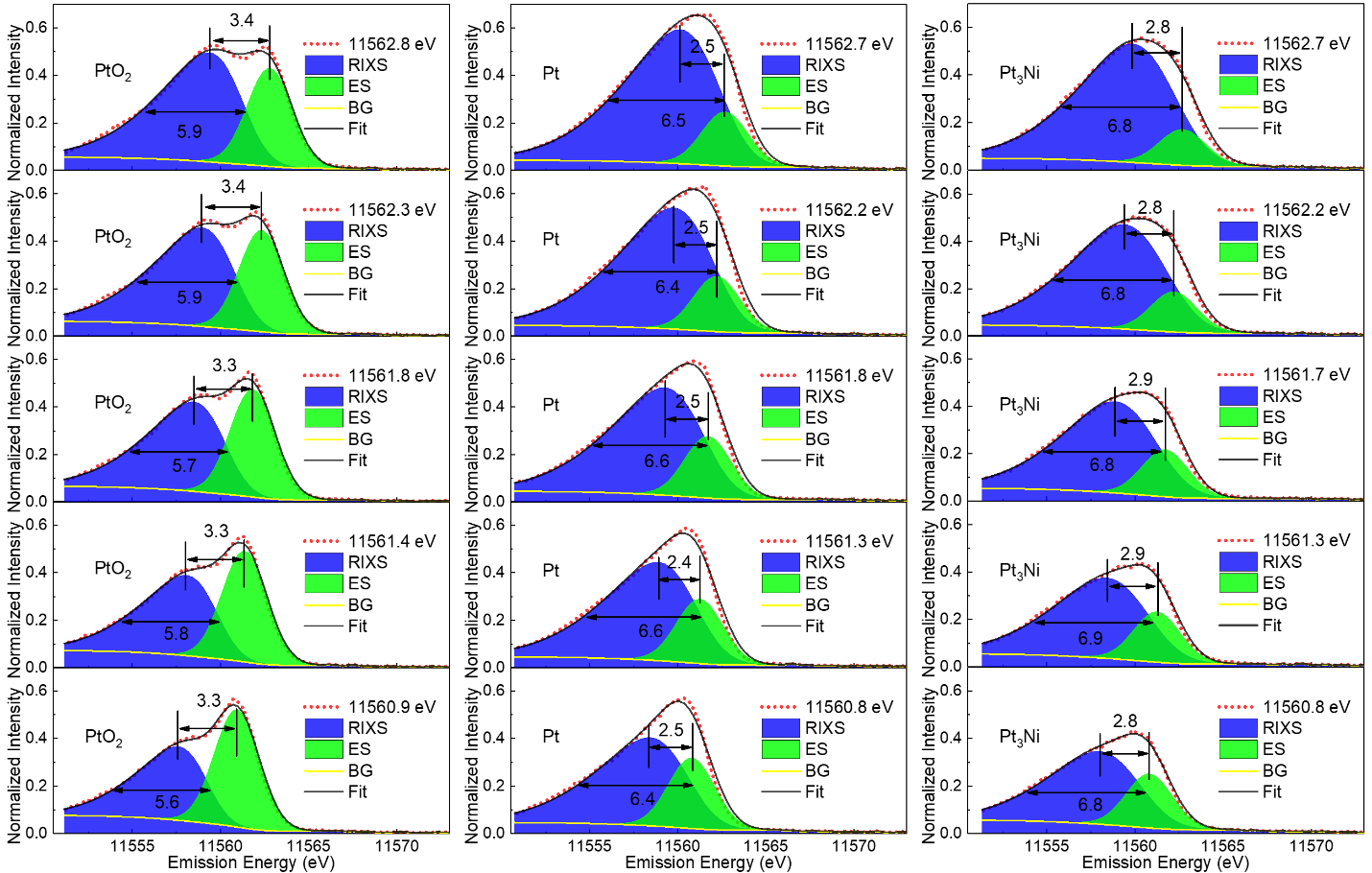


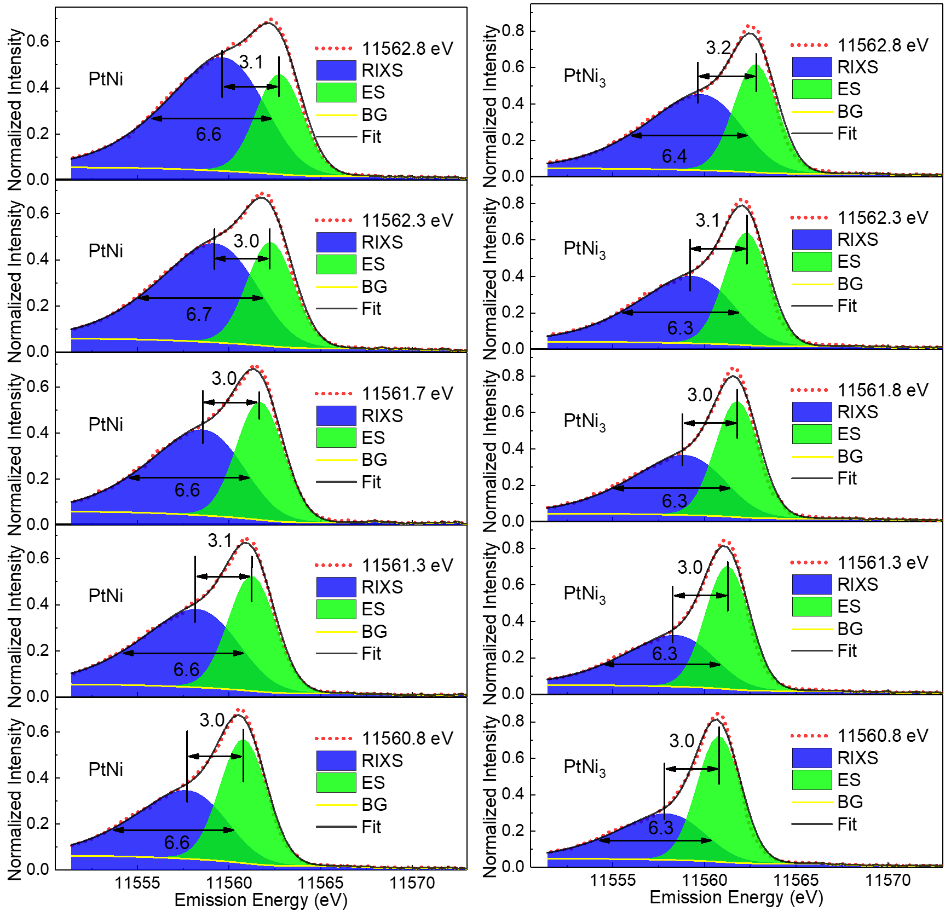


Figure S5. Fitting results of RIXS below resonance. Five spectra (excitation energies at 11560.8~11562.8 eV) from each sample are fitted with an asymmetric peak with the ES (Gaussian peak with the width for each sample extracted in Figure S4). The Shirley background (BG) is combined with the asymmetric peak to provide the RIXS signal.

**Reference**

1. Chen J*, et al.* Elucidating the many-body effect and anomalous Pt and Ni core level shifts in X‑ray photoelectron spectroscopy of Pt−Ni alloys. *J Phys Chem C* **124**, 2313-2318 (2020).
